# Attenuation of Knee Osteoarthritis Progression in Mice through Polarization of M2 Macrophages by Intra-Articular Transplantation of Non-Cultured Human Adipose-Derived Regenerative Cells

**DOI:** 10.3390/jcm10194309

**Published:** 2021-09-22

**Authors:** Kohei Kamada, Takehiko Matsushita, Takahiro Yamashita, Tomoyuki Matsumoto, Hideki Iwaguro, Satoshi Sobajima, Ryosuke Kuroda

**Affiliations:** 1 Department of Orthopaedic Surgery, Kobe University Graduate School of Medicine, Kobe 650-0017, Japan; koheikamada1202@gmail.com (K.K.); y.kidai.83716@gmail.com (T.Y.); matsun@m4.dion.ne.jp (T.M.); kurodar@med.kobe-u.ac.jp (R.K.); 2 Sobajima Clinic, Higashiosaka, Osaka 577-0011, Japan; iwaguro@soba-cli.com (H.I.); orthohealing@soba-cli.com (S.S.)

**Keywords:** adipose-derived regenerative cells, stromal vascular fraction, osteoarthritis, macrophage

## Abstract

Adipose-derived regenerative cells (ADRCs) are non-cultured heterogeneous or mixed populations of cells obtained from adipose tissue by collagenase digestion. The injection of ADRCs have been tried clinically for the treatment of osteoarthritis (OA). The purpose of this study was to evaluate the effect of intra-articular transplantation of human ADRCs on OA progression in mice and the effect of ADRCs on macrophage polarization. In in vivo experiments, BALB/c-nu mice with knee OA received intra-articular transplantation of either phosphate buffered-saline or human ADRCs. OA progression was evaluated histologically and significantly attenuated in the ADRC group at both four and eight weeks postoperatively. The expression of OA-related proteins in the cartilage and macrophage-associated markers in the synovium were examined by immunohistochemistry. The numbers of MMP-13-, ADAMTS-5-, IL-1β-, IL-6- and iNOS-positive cells significantly decreased, and type II collagen- and CD206-positive cells were more frequently detected in the ADRC group compared with that in the control group. In vitro co-culture experiments showed that ADRCs induced macrophage polarization toward M2. The results of this study suggest that the intra-articular transplantation of human ADRCs could attenuate OA progression possibly by reducing catabolic factors in chondrocytes and modulating macrophage polarization.

## 1. Introduction

Osteoarthritis (OA) of the knee is a common disease and one of the major causes of joint dysfunction and physical disability in the elderly [1]. The pathogenesis of OA involves wear of the articular cartilage and associated cartilage degeneration. Although surgical treatments including total knee arthroplasty and high tibial osteotomy are performed to improve pain and joint function in patients with advanced-stage OA, most patients with OA are often treated conservatively. Since articular cartilage is an avascular tissue and poor in cellular components, drug accessibility, and its self-healing capacity are relatively low. Due to this unique tissue structure, treatment of OA is challenging, and to date, disease-modifying drugs for OA have not been developed.

In recent years, regenerative medicine based on stem cells has attracted increasing attention. Adipose tissue is a source of stem cells and contains mesenchymal adipose-derived stem cells (ADSCs), which have been shown to undergo adipogenic, osteogenic, chondrogenic, neurogenic, and myogenic differentiation in vitro [2]. Adipose tissue is relatively easy to harvest by liposuction and contains an abundance of stem cells, wherein approximately 5% of adipose tissue-derived nucleated cells have characteristic stem cell phenotypes; in contrast, only 0.0001–0.01% of bone marrow-derived mesenchymal cells show stem cell properties [2,3]. As a result, intra-articular injection of ADSCs has been used for the treatment of OA [4,5,6,7,8]. However, the cost of cell culture, two-step surgery, cell harvest, and implantation impede its widespread use in clinical settings.

Adipose-derived regenerative cells (ADRCs) comprise a non-cultured heterogeneous or mixed population of cells found in the stromal vascular fraction (SVF) obtained after collagenase digestion of adipose tissue [9,10]. Previous clinical studies have shown that implantation of ADRCs could be useful in the treatment of chronic ulcers of the lower limbs [11], ischemic cardiomyopathy [12], fat grafting [13], and limb ischemia [14]. Moreover, the therapeutic effects of injected non-cultured adipose-derived SVF for treating OA have been investigated in clinical trials, and an improvement in OA symptoms was reported [15,16,17,18]. While the inhibitory effects of the intra-articular injection of non-cultured adipose-derived SVF on OA progression were suggested in some animal studies [19,20], the effects of human SVF on OA progression and its mechanisms have not been fully elucidated.

In recent years, the involvement of macrophages in the synovium as a mechanism for the development and suppression of OA has been attracting attention [21,22,23]. Macrophages are plastic cells that are classified as classically activated M1 macrophages or alternatively activated M2 macrophages [24]. M1 macrophages, which are activated by interferon-γ (IFN-γ), lipopolysaccharide (LPS), and tumour necrosis factor alpha (TNF-α), are known to release inflammatory cytokines that promote tissue damage and inflammation [21,23,25,26]. M2 macrophages, which are induced by interleukin-4 (IL-4) and IL-13, are also known as wound-healing macrophages to release anti-inflammatory cytokines that promote tissue remodelling and suppress inflammation [21,25,27]. The polarized synovial macrophages appear to be a suitable therapeutic target for the early prevention and treatment of OA. Several in vitro studies have reported the promotion of M2 macrophages by platelet-rich plasma (PRP) [28] and mesenchymal stem cells [29,30,31,32]. However, few studies have evaluated the effect of human SVF or ADRCs on macrophage polarization.

Therefore, the purpose of this study was to evaluate the therapeutic effect of intra-articular transplantation of ADRCs on OA progression and to elucidate the effect of ADRCs on macrophage polarization, a potential mechanism of the therapeutic effects of ADRC.

## 2. Materials and Methods

### 2.1. Preparation of ADRCs

ADRCs were obtained from 10 patients who received intra-articular injection of ADRCs for the treatment of knee pain. Post injection, the remaining fraction of cells was used for our experiments under the patients’ consent. Patients underwent a liposuction procedure under general anaesthesia to obtain 100–360 mL of adipose tissue. SVF cells were extracted from the patients’ abdominal or subcutaneous fat using the Celution^®^ 800/CRS system (Cytori Therapeutics Inc., San Diego, CA, USA), which consists of two parts: one for tissue washing and digestion and the other for concentrating cells, according to the manufacturer’s instructions. Briefly, the adipose tissue was washed to remove blood and debris. Celase^®^ GMP, a mixture of highly purified collagenase and neutral protease enzymes, was added and incubated at ~37 °C for 20 min, with continuous mixing, to digest the aspirated adipose tissue. After digestion, the SVF cells were concentrated by centrifugation and washed to remove the Celase^®^ reagent. Next, SVF cells were extracted from the system and counted to prepare a specified dose in 5 mL of lactated Ringer’s solution. The whole system was operated aseptically using clinical-grade solutions such as saline and lactated Ringer’s, and single-use Celution TM consumables. The SVF cell count and viability were determined at each investigational site using the NC-100 TM NucleoCounter^®^ Automated Cell Counting System (Chemometec, Allerod, Denmark). Patient (donor) information, including the harvest site and cell numbers, is listed in Table 1. Approval for ADRCs extraction from patients was obtained from the ethics committee of our institution (Approval number P150803). The ADRCs obtained from the patients were used for in vivo or in vitro experiments on the same day.

### 2.2. In Vivo Experiment

#### 2.2.1. Establishment of the Experimental OA Model

BALB/c-nu mice purchased from Japan SLC, Inc. (Shizuoka, Japan) were used in this study (total 66 mice, all male). Mice were anaesthetised via intraperitoneal injection of ketamine (100 mg/kg) and the right knee joints were exposed using the medial parapatellar approach. Experimental OA was induced by resecting the medial meniscotibial ligament under a microscope to destabilise the medial meniscus in the knee joint of 12-week-old mice [33]. ADRCs were transplanted at a dose of 2.0 × 10^4^ cells/6 µL/knee (ADRC group). The dose of the cells was determined based on previous studies by adjusting the number of cells as per the bodyweight [19,34,35]. For the control group, phosphate-buffered saline (PBS) was administered (6 µL/knee). Littermates were divided across both groups. The knee joint capsule and skin were closed using 6–0 nylon sutures. Mice were maintained under pathogen-free conditions and allowed free access to food and water. Mice were sacrificed at four and eight weeks post-surgery (n = 3 mice per group for each time point and each donor). Body weight of each mouse was measured at the time of surgery and sacrifice (Table 2). There was no significant difference between the 2 groups. For the analysis of the migration of the transplanted cells, mice (n = 3 mice for each group) were sacrificed one week after implantation. All mice were sacrificed with intraperitoneal injection of ketamine and cervical dislocation. All procedures were performed after obtaining approval from the Institutional Animal Care and Use Committee at our institution (approval number P180103).

#### 2.2.2. Histological Analysis

The entire right knee joint was fixed overnight at 4 °C in 4% paraformaldehyde prepared in 0.1 M PBS. Decalcification was performed for 2 weeks with 10% ethylenediaminetetraacetic acid, after which the specimens were embedded in paraffin wax. Each specimen was cut into 6-μm thick sections along the sagittal plane. For hematoxylin and eosin staining, sections were stained with hematoxylin for 4 min, followed by eosin staining for 1.5 min. For safranin O and fast green double staining, sections were stained with hematoxylin for 4 min, followed by fast green staining for 5 min, and safranin O staining for 1 min. Three sections each were selected from the medial femoral condyle and medial tibial plateau, and images were obtained at 40× magnification. The histological OA grade for each field was evaluated using the OARSI cartilage OA histopathology-grading system (scores ranging from 0–6) [36]. OA grading was assessed by a single observer (TY), who was blinded to the study groups.

#### 2.2.3. Immunohistochemistry

Deparaffinised sections were digested with proteinase (Dako Denmark A/S, Glostrup, Denmark) for 10 min and treated with 3% hydrogen peroxide (Wako Pure Chemical Industries Ltd., Osaka, Japan) to block endogenous peroxidase activity. After antigen retrieval, the sections were incubated overnight at 4 °C with primary antibodies against the following mouse proteins: matrix metalloproteinase-13 (MMP-13, 1:100, Abcam, Cambridge, UK), A Disintegrin And Metalloproteinase with Thrombospondin motifs-5 (ADAMTS-5, 1:100, Santa Cruz Biotechnology Inc., Santa Cruz, CA, USA), IL-6 (1:100, Abcam), type II collagen (1:100, Abcam), IL-1β (1:100, Abcam), inducible nitric oxide synthase (iNOS, 1:100, Abcam), Cluster of Differentiation 206 (CD206, 1:100, Abcam), and human nuclear antigen (1:100, Abcam). Sections were then incubated with peroxidase-labelled anti-rabbit or mouse immunoglobulin (Histofine Simple Stain MAX Po; Nichirei Bioscience, Tokyo, Japan) at room temperature for 30 min. Staining was visualised using the peroxidase substrate, 3,3′-diaminobenzidine, which resulted in the development of a brown-coloured signal, with methyl green used for counterstaining. As negative controls, a non-immune mouse or rabbit immunoglobulin (1:50 dilution) was used instead of the primary antibodies. Images were acquired using a Biozero microscope (Keyence Corp., Itasca, OH, USA).

### 2.3. In Vitro Experiment

#### 2.3.1. Preparation and Culture of Macrophages

Murine macrophage-like J774.1 cells were obtained from the RIKEN Bioresource Center (Ibaraki, Japan). Cells were cultured in RPMI-1640 medium (Thermo Fisher Scientific, Gibco^®^, Waltham, MA, USA) containing 10% heat-inactivated fetal bovine serum (FBS) (Sigma; St. Louis, MO, USA) in an incubator at 37 °C and 5% CO_2_. J774.1 cells were used as confluent monolayers at passages 3 through 5.

#### 2.3.2. Co-Culture Assay and Grouping

To confirm the paracrine effect of ADRCs on macrophage polarization, a trans-well co-culture system (Cell Culture Insert plates; Thermo Fisher Scientific, Tokyo, Japan) was used. In this system, macrophages were seeded in the base of 12-well plates and ADRCs were seeded on the insert. Thus, only factors secreted from the cells would pass the membrane. To induce M1 polarization, 50 ng/mL IFN-γ (RayBiotech, Norcross, GA, USA) and 100 ng/mL LPS (Sigma-Aldrich, St. Louis, MO, USA) were added to the basal medium, and 20 ng/mL IL-4 (RayBiotech) was added to induce M2 polarization under the following conditions.

**Figure 1:**
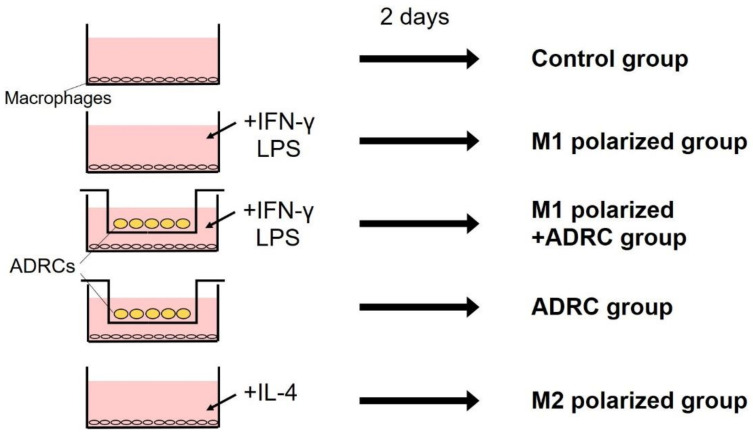
Summary of in vitro experiment. Macrophages were then divided into five groups and cultured for 2 days under the following conditions: control group, basal medium only; M1-polarized group, IFN-γ/LPS stimulation; M1-polarized + ADRC group–IFN-γ/LPS stimulation + co-culture with ADRCs; ADRC group, co-culture with ADRCs; M2-polarized group, IL-4 stimulation.

First, macrophages were seeded at a density of 1 × 10^5^ cells/well in the aforementioned 12-well plates and cultured for two days. Macrophages were then divided into five groups and cultured for two days under the following conditions: control group, basal medium only; M1-polarized group, IFN-γ/LPS stimulation; M1-polarized + ADRC group–IFN-γ/LPS stimulation + co-culture with ADRCs (2 × 10^4^ cells/well); ADRC group, co-culture with ADRCs (2 × 10^4^ cells/well); M2-polarized group, IL-4 stimulation. A summary of the grouping is presented in Figure 1.

#### 2.3.3. Immunocyto-Fluorescent Staining

To visualise macrophage polarization, immunofluorescent staining for iNOS (M1-like macrophage marker) and CD206 (M2-like macrophage marker) were performed as previously described [37]. After washing with PBS and formalin fixation, the cells on the slides were incubated with the conjugated primary antibodies iNOS (1:100, bs-2072R-FITC, Bioss, Woburn, MA, USA) and CD206 (1:100, bs4727R-Cy5.5, Bioss) for 3 h at 37 °C. The slides were washed with PBS three times. Nuclei were then counterstained blue with 4′,6-diamidino-2-phenylindole (DAPI) for 15 min at room temperature. Images were obtained using a Biozero microscope. iNOS- and CD206-positive cells were fluoresced green and red, respectively. Experiments were performed using ADRCs from four donors and were repeated three times for each donor.

### 2.4. Statistical Analysis

Statistical analysis was performed using GraphPad Prism 9 (GraphPad Software, San Diego, CA, USA). The data obtained were tested for normality of distribution through the Kolmogorov–Smirnov test. When the data were normally distributed, one-way ANOVA test was performed and Tukey–Kramer test was used as a multiple comparison test. When data were rejected, the Kruskal–Wallis test was performed and the Bonferroni–Dunn test was used as a multiple comparison test to compare differences among groups. The significance level was set at *p* < 0.05.

## 3. Results

### 3.1. Histological Evaluation of OA Progression

**Figure 2:**
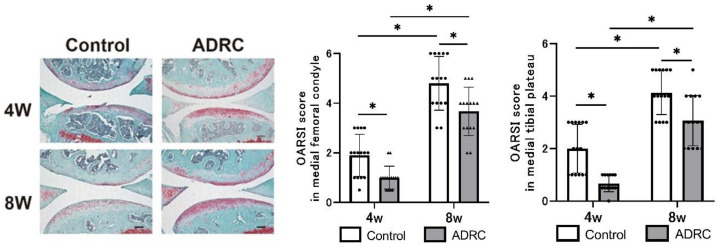
Histological analysis of osteoarthritis progression. Representative images at four and eight weeks after transplantation. Images were taken at 200×. Asterisk indicates statistically significant differences in multiple-comparison testing. Scale bars = 100 µm.

OA progression was observed in both groups; however, the histopathological score, based on the Osteoarthritis Research Society International (OARSI) OA histopathology-grading system, of the medial femoral condyle and tibial condyle in the ADRC-treated group was significantly lower than that in the control group at both four and eight weeks post-surgery (Figure 2).

### 3.2. Immunohistochemical Analyses of OA-Related Markers in the Articular Cartilage

**Figure 3:**
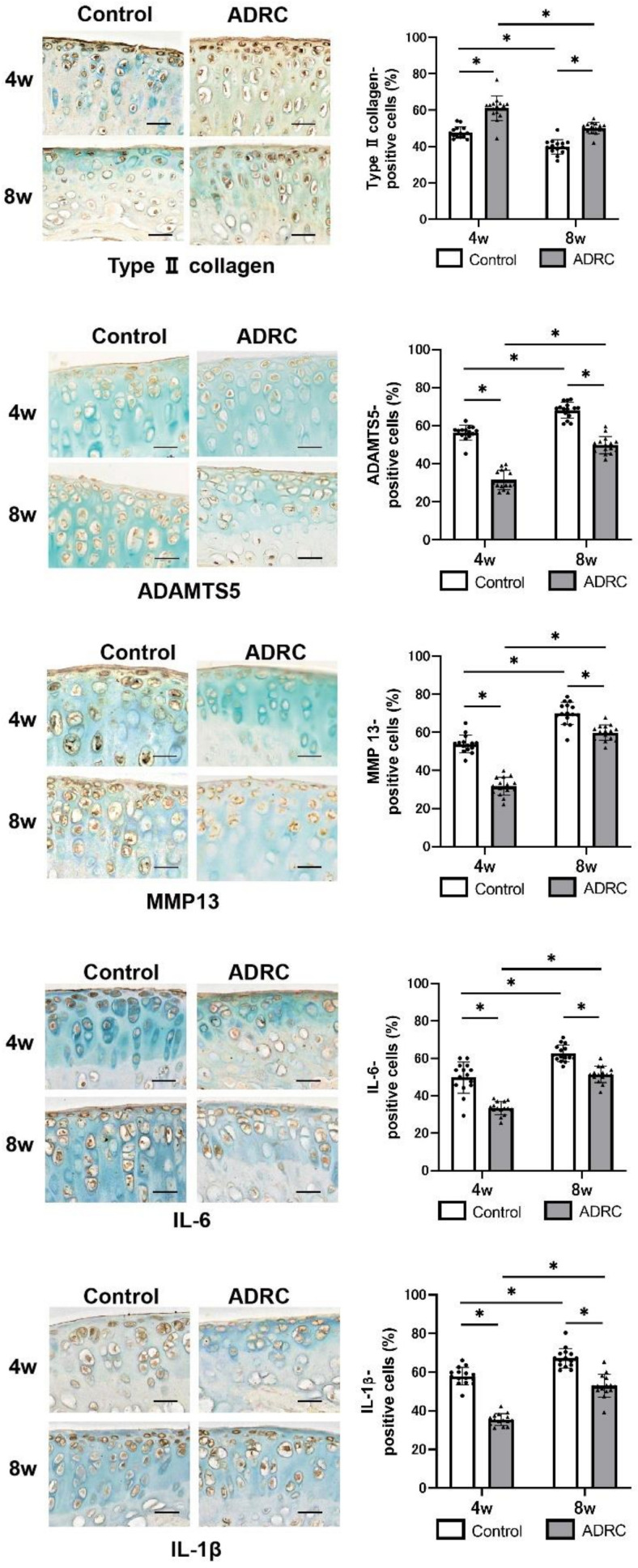
Immunohistochemical analyses of type II collagen, cartilage degrading enzymes, and inflammatory cytokines. Representative images at four and eight weeks after transplantation. Graphs show the ratio of the number of positive cells to the number of total cells for each protein at four and eight weeks after transplantation. Asterisk indicates a statistically significant difference. Scale bars = 50 µm.

Immunohistochemical analysis showed that the number of type II collagen-positive cells was significantly higher, whereas the number of MMP-13-, ADAMTS-5-, IL-6-, and IL-1β-positive chondrocytes were significantly lower, in the ADRC-treated group compared with that in the control group at both four and eight weeks post-surgery (Figure 3).

### 3.3. Migration of Transplanted ADRCs

**Figure 4:**
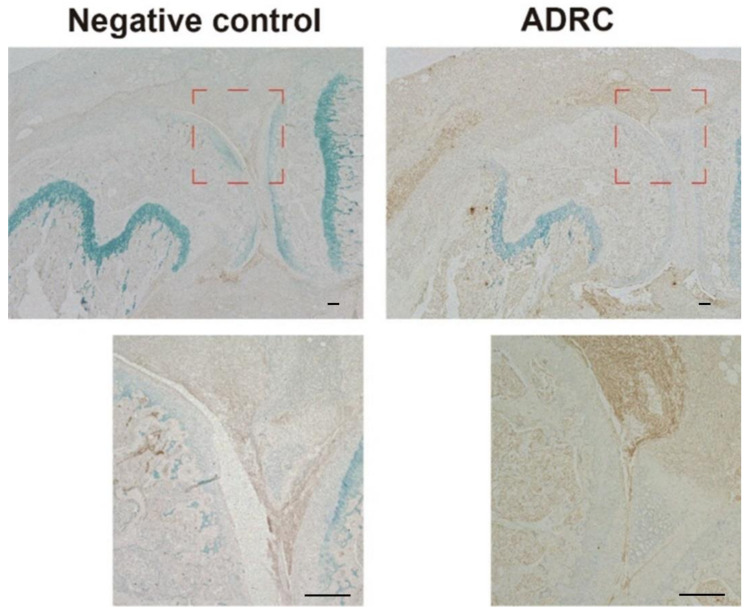
Immunohistochemical analyses of human nuclear antigen one week after transplantation. Representative images are shown. Lower panel show the magnified images correspond to the square area on the upper images. Scale bars = 100 µm.

To assess the fate of the transplanted ADRCs, immunohistochemical analysis of the human nuclear antigen was performed. Human nuclear antigen was mainly observed in the synovial tissue of the supra-patellar pouch and infra-patellar fat pad area one week after transplantation. However, it was not detected four weeks after transplantation. Very low levels of staining were detected in the superficial layer of the meniscus one week after transplantation but not in the cartilage at any time point (Figure 4).

### 3.4. The Effects of ADRCs on the Synovium

**Figure 5:**
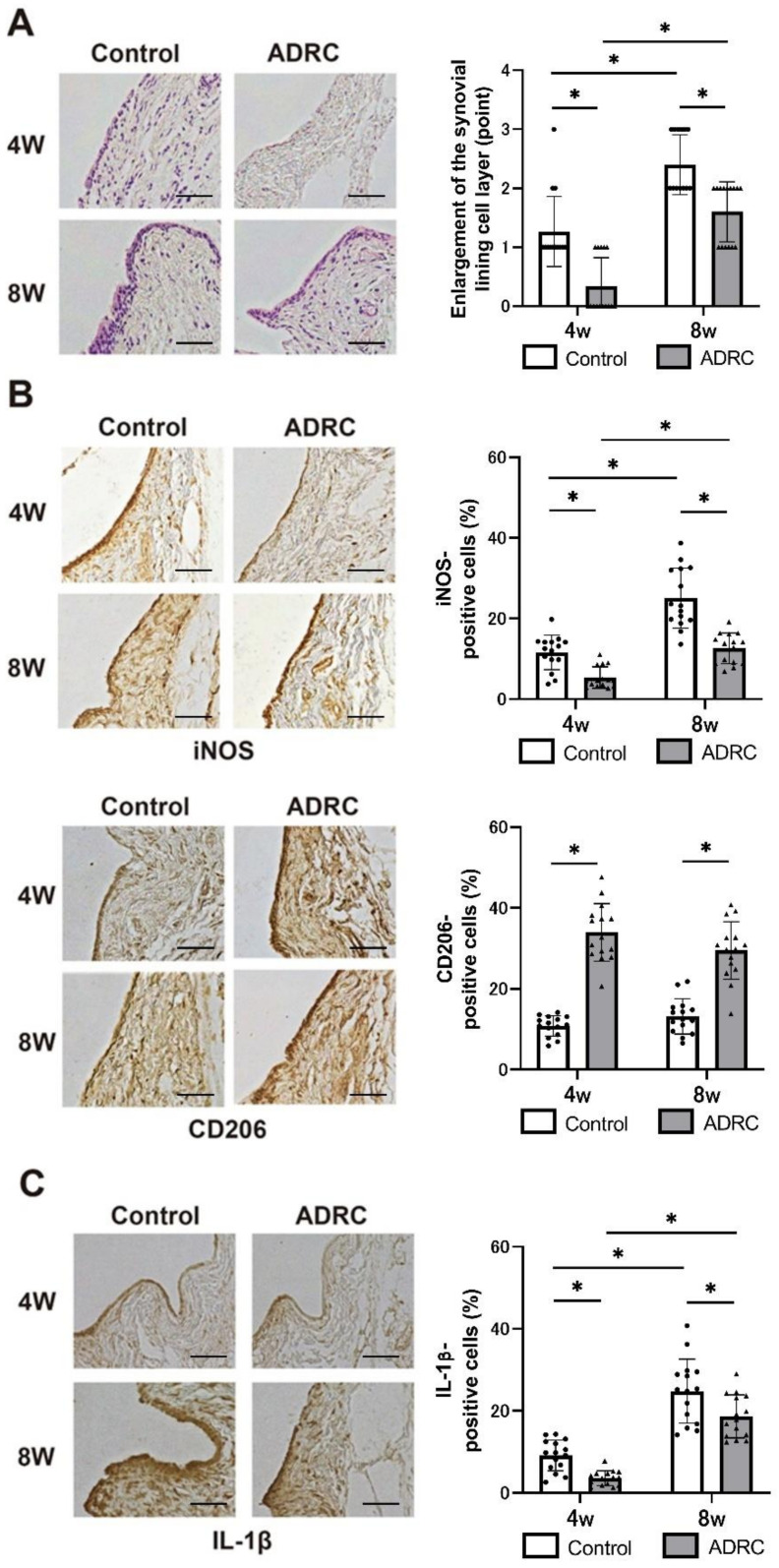
Histological and immunohistochemical analyses of the synovium. (**A**) Hematoxylin and Eosin staining. Images were taken at 100×. (**B**) Immunohistochemistry results for iNOS and CD206. (**C**) Immunohistochemistry results for IL-1β. Graphs show the results of the synovial lining cell layer and the ratio of the positive cells to the total cells for each protein at four and eight weeks after transplantation. Asterisk indicates a statistically significant difference. Grading of enlargement of the synovial lining cell layer was based on the method of previous report [38]. Scale bars = 100 µm.

The cell layer lining the synovium in control mice was thicker than that in the mice treated with ADRCs (Figure 5A). To examine the effects of ADRCs on synovial macrophages, we performed immunohistochemical analysis for macrophage markers. In the synovium, the ratio of iNOS (an M1-like macrophage marker)-positive cells was higher in the control group than that in the ADRC-treated group, whereas the ratio of CD206 (an M2-like macrophage marker)-positive cells was higher in the ADRC-treated group than that in the control group (Figure 5B). In addition, a higher ratio of IL-1β-positive cells was observed in the control group than that in the ADRC group (Figure 5C).

### 3.5. Immunocyto-Fluorescent Analysis

**Figure 6:**
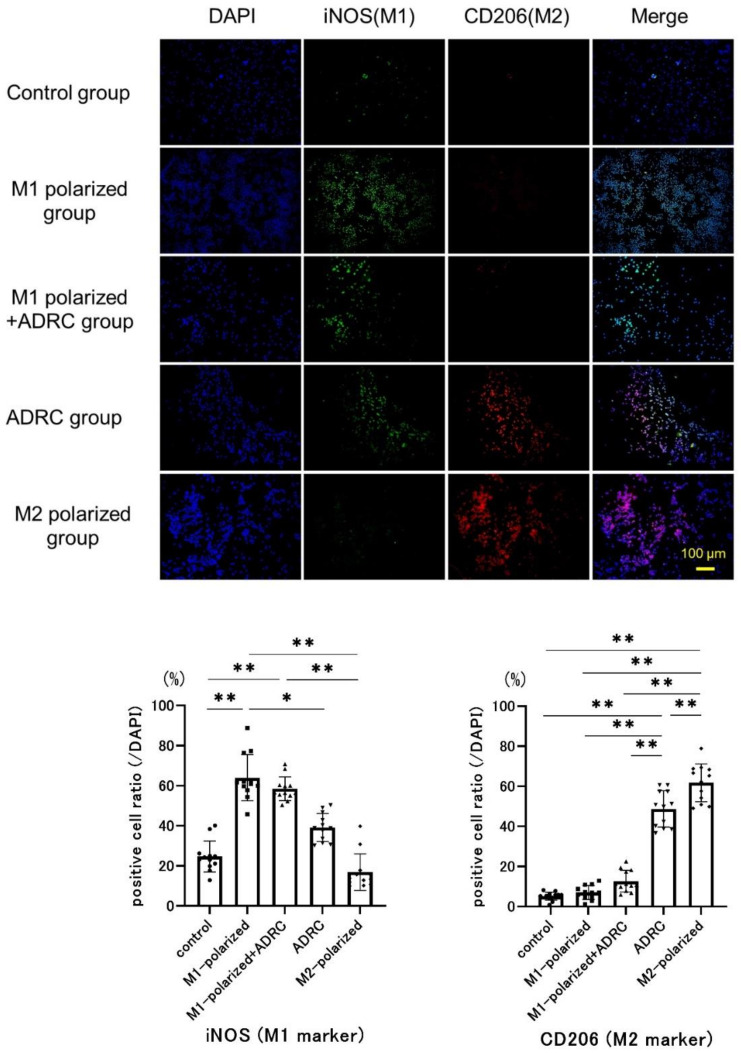
Immunocyto-fluorescent analysis of the effect on the macrophages by co-culture assay with ADRCs. Representative images showed immunofluorescent staining for the macrophage marker iNOS (green) and CD206 (red). Scale bars = 100 μm. Graphs show the ratio of the number of positive cells to the number of DAPI-positive cells for iNOS and CD206. * *p* < 0.05, ** *p* < 0.01.

The effect of ADRCs on macrophage polarization was evaluated by immunofluorescence staining (Figure 6). The ratio of iNOS-positive cells in the M1-polarized + ADRC group (58.5 ± 6.0%) tended to be lower than that in the M1-polarized group, but the difference was not significant. The ratio of CD206-positive cells in the ADRC group (48.9% ± 9.1%) was significantly higher than that in the control group (5.1 ± 2.1%), M1-polarized group (7.0 ± 3.5%), and M1-polarized + ADRC group (12.6% ± 5.4%) at *p* < 0.01. The ratio of CD206-positive cells in the M1-polarized + ADRC group tended to be higher than that in the M1-polarized group, but the difference was not significant.

## 4. Discussion

The primary finding of this study was that intra-articular transplantation of human ADRCs attenuated OA progression in a mouse model of OA and that the ability of ADRCs to polarize macrophages to M2 may have contributed to the OA suppression mechanism. In addition, the transplanted cells migrate to the synovium of the joint and act on synovial macrophages, which may play a role in suppressing the production of cartilage-degrading enzymes and inflammatory cytokines.

Despite a recent increase in the number of clinical studies investigating the benefits of autologous SVF transplantation in OA treatment, fundamental research studies on the effects of autologous SVF transplantation on OA progression are sparse. Kuroda et al. reported that intra-articular transplantation of uncultured adipose-derived SVF attenuated OA progression in a rabbit model of anterior cruciate ligament transection (ACLT). In addition, co-culturing chondrocytes from ACLT rabbits with SVF in the absence of physical contact, using a trans-well system, resulted in higher cell viability and increased expression of anabolic markers, such as SOX 9 and COL2, and a lower expression of MMP-13 compared with that in chondrocytes cultured without SVF. These results suggest that soluble factors from SVF can exert protective effects on chondrocytes and contribute to improving OA symptoms [20].

Similar to our study, Dykstra et al. examined the effects of intra-articular injection of human adipose tissue-derived SVF and mesenchymal stem cells (MSCs) on the progression of cartilage damage using a cartilage injury model based on nonobese diabetic/severe combined immunodeficiency mice [39]. They reported that both SVF and MSCs attenuated the progression of cartilage injury, although details including time of evaluation were not described. In the present study, we showed that transplantation of ADRCs significantly attenuated OA progression in a mouse model of OA at four and eight weeks postoperatively. We believe that reduction in the levels of cartilage-degrading enzymes, such as ADAMTS5 and MMP13, and inflammatory cytokines, such as IL-1β and IL-6, underlies the mechanism responsible for attenuating OA progression in ADRC-treated mice.

The fate of transplanted cells is one of the intriguing aspects that should be considered to elucidate the beneficial effects of cell transplantation. Some studies have described the migration and fate-specification of cells injected or transplanted in the joint [40]. For example, Sato et al. reported that bone marrow-derived human mesenchymal stem cells labeled with carboxyfluorescein diacetate succinimidyl ester were detected within the cartilage, meniscus and synovium in the guinea pigs that exhibit spontaneous development of OA, although the evaluated timing was not addressed. They also reported that the labeled cells with the cartilage were detected at three-to-five-weeks post-transplantation [41]. Similarly, Li et al. developed a new cell tracking method using DiD, an analog of dialkylcarbocyanine, to investigate the cell fate after they were articularly injected [40]. They labelled human ADSCs with DiD and injected them into the knee joint of the rat after medial meniscectomy. Human ADSCs visualised using a human mitochondrial antibody were detected in the meniscus and cartilage. Interestingly, human Ki67-positive cells were detected for up to 10 weeks after injection, suggesting that the integrated cells proliferated in the region. These reports also suggest that the cultured mesenchymal cells could migrate into the cartilage regardless of the origin of the cells. Unlike the results of Sato et al. and Li et al., in our study, we did not detect the integration of the transplanted cells into cartilage at one or even four weeks after transplantation. This difference could be attributed to the difference in evaluation times or sensitivity of detection methods. Desando et al. also examined cell migration patterns after intra-articular injection of fluorescence-labeled ADSCs, SVF, and micro-fragmented adipose tissue (MFAT) in a rabbit model of ACLT [42]. They detected the migration of ADSCs and SVF into the synovium seven days after injection, while migration of the cells from MFAT was observed in the cartilage. Thirty days after the injection, ADSCs were observed mainly in the meniscus, SVF was observed in the meniscus and cartilage, and labelled cells from the MFAT were mainly observed in the synovium. Although the migration patterns were different depending on the processing of the adipose tissue-derived cells. Collectively, the results suggest that the transplanted cells mainly attach to the synovium in the early phase and later migrate into the cartilage.

In the present study, immunohistochemical analysis suggested that the transplanted cells migrated into the synovium. Furthermore, we found that the number of CD206-positive M2-like macrophages increased while that of iNOS-positive M1-like macrophages decreased in the synovium. Recent studies have implicated macrophages to play an important role in the pathogenesis of OA [22,37,43]. Particularly, an increased M1/M2 macrophage ratio associated with elevated expression of inflammatory cytokines and MMPs was suggested as a cause of OA [21]. Therefore, immunomodulation via macrophage polarization upon ADRCs transplantation may have contributed to the attenuation of OA in ADRC-treated mice.

To examine the effects of ADRCs on macrophage phenotype, we conducted a co-culture experiment using the trans-well system. Co-culturing with ADRCs significantly increased the ratio of macrophages that were positive for the M2 maker CD206, whereas it did not significantly change under the condition of M1 induction. Meanwhile, co-culturing with ADRCs did not significantly affect the ratio of macrophages that were positive for the M1 marker iNOS, regardless of M1 induction. These results suggested that ADRCs could promote phenotypic changes in macrophages toward M2 like phenotype. Since the trans-well system allows only exchange of soluble factors between the ADRCs and macrophages, the results suggest that soluble factors from ADRCs promote phenotypic changes of macrophages toward an M2-like phenotype. Ragni et al. examined secreted cytokines and extracellular vesicles containing miRNAs from ADSCs and identified miRNAs that possibly modulate macrophage polarization [44]. Zhao et al. showed that exosomes from ADSCs transferred into macrophages in a co-culture experiment in which treatment with ADSC-derived exosomes induced M2 like-gene expression changes in macrophages [32]. These previous findings suggest that exosomes from ADRCs mediate the induction of M2 macrophage polarization by ADRCs.

Regarding the M1 phenotype, ADRCs-mediated prevention of the phenotype was not observed in the co-culture experiment. Rather a non-statistically significant increase of M1 marker expression was observed while statistically significant increased M2 marker expression was observed after coculture with ADRCs. These observations appear to be inconsistent with the in vivo observation. However, during the early phase of differentiation, both M1 and M2 marker expression could be once upregulated [45] before polarizing toward either phenotype. In this study, we used immature bone marrow macrophage and evaluated the differentiation status 2 days after co-culture with ADRCs. Therefore, the slight upregulation of the M1 marker expression may reflect a response in the early phase of differentiation although the differentiation were more prone toward M2. 

Similar to our results, Kudlik et al. demonstrated that mouse bone marrow-derived cells have a significant polarizing effect from unpolarized macrophages to M2 in a co-culture system, whereas the cells had no significant effect on M1–M2a and M1–M2b polarization [30]. Therefore, the effects of ADRCs on macrophage phenotype could be limited to the induction of the M2 phenotype from an unpolarized state. However, it is possible that the condition of M1 induction in the culture system was more potent than the actual in vivo condition to detect the effects on M1–M2 polarization. Further studies are required to elucidate the mechanism of macrophage regulation by ADRCs.

Our study had a few limitations. First, ADRCs transplantation was performed in mice at the time of OA induction, and therefore, the protective effects of ADRCs against advanced OA were not evaluated. Second, a single dose of ADRCs was used in this study, and the optimal dosage remains to be determined. Third, ADRCs were obtained from a relatively small number of patients and we did not investigate the heterogeneity of donors. Although we did not observe any obvious differences among the patients, the efficacy of ADRCs obtained from a larger number of patients needs to be determined to understand whether patient background affects the therapeutic benefits of intra-articular ADRCs transplantation on OA progression. Fourth, we did not examine the status of the macrophage phenotypes and OA-related markers at the timing when ADRCs still existed in the joint. Therefore, how ADRCs in the synovium affect surrounding cells remain unknown.

## 5. Conclusions

Intra-articular transplantation of human ADRCs attenuated OA progression in a mouse model of OA possibly by modulating macrophage polarization by soluble factors secreted from ADRCs and consequently reducing catabolic factors in chondrocytes. Our study outcomes propose that ADRCs can be used for the treatment of OA in the future.

## Figures and Tables

**Table 1:** Patient information. Cells from patients #1 to #6 were used for in vivo experiments, and cells from patients #7 to #10 were used for in vitro experiments.

Patient	Age	Sex	BMI	Harvest Site	Cell Number	Viability
#1	63	Female	23.7	Abdominal	1.9 × 10^6^	88.3%
#2	53	Male	30.9	Thigh	3.9 × 10^6^	96.2%
#3	62	Female	24.5	Buttock	2.5 × 10^6^	92.3%
#4	70	Female	23.2	Buttock	3.5 × 10^6^	92.8%
#5	72	Female	25.6	Buttock	2.8 × 10^6^	94.2%
#6	70	Female	24.3	Buttock	2.1 × 10^6^	91.8%
#7	69	Male	26.8	Buttock	1.6 × 10^6^	84.8%
#8	60	Female	23.9	Buttock	2.4 × 10^6^	91.6%
#9	69	Female	22.2	Buttock	2.1 × 10^6^	88.7%
#10	69	Female	25.4	Buttock	4.3 × 10^6^	93.8%

**Table 2:** Mean body weight (g) of mice in the control and ADRC groups at the indicated time points.

	At Surgery	4 Weeks	8 Weeks
**Control Group**	20.8 ± 2.4 g	21.9 ± 1.9 g	23.2 ± 2.6 g
**ADRC Group**	20.6 ± 1.3 g	21.4 ± 1.2 g	22.8 ± 1.8 g

## Data Availability

The data presented in this study are available on request from the corresponding author. The data are not publicly available because of confidentiality issues.
